# Optimal protein concentration in diets for sows during the transition period

**DOI:** 10.1093/jas/skae082

**Published:** 2024-03-22

**Authors:** Jakob C Johannsen, Martin T Sørensen, Peter K Theil, Thomas S Bruun, Chantal Farmer, Takele Feyera

**Affiliations:** Department of Animal and Veterinary Sciences, Aarhus University, Campus Viborg, DK-8830 Tjele, Denmark; Department of Animal and Veterinary Sciences, Aarhus University, Campus Viborg, DK-8830 Tjele, Denmark; Department of Animal and Veterinary Sciences, Aarhus University, Campus Viborg, DK-8830 Tjele, Denmark; SEGES Innovation, DK-8200 Aarhus N, Denmark; Agriculture and Agri-Food Canada, Sherbrooke Research and Development Centre, Sherbrooke, QC, CanadaJ1M 0C8; Department of Animal and Veterinary Sciences, Aarhus University, Campus Viborg, DK-8830 Tjele, Denmark

**Keywords:** Colostrum, dietary protein, lysine, milk yield, sow nutrition, transition period

## Abstract

The aim of the present study was to determine the optimal concentration of dietary protein required in transition diets for multiparous sows that enhance the farrowing process, colostrum production, and subsequent lactation performance. Forty-eight multiparous sows were allotted to one of six dietary treatments according to body weight (290 ± 3 kg) and parity (3.8 ± 0.2) from day 108 of gestation until 24 h after the onset of farrowing. The diets were isoenergetic and contained increasing concentrations of dietary protein (expressed as standardized ileal digestible [**SID**] Lys) and were supplied at a daily feed supply of 3.8 kg. On day 108 of gestation and days 2, 7, 14, 21, and 28 of lactation, body weight, and back fat thickness were recorded, and blood was sampled on day 108 of gestation, at the onset of farrowing, and days 3, 10, 17, and 24 of lactation from the sows for analysis of plasma metabolites. On day 115 of gestation, urine, and feces were collected for nitrogen (**N**) balance. The number of liveborn and stillborn piglets and time of birth were recorded and blood from every fourth piglet was sampled at birth for blood gas analysis. Piglets were weighed individually from birth until weaning, to estimate the colostrum and milk yield of the sows. Colostrum and milk samples were collected, and their compositions were determined. On days 3 and 28 of lactation, sows were injected with deuterium oxide to estimate body composition. The N utilization was maximized when the concentration of SID Lys in the transition diet was 6.06 g/kg (*P* < 0.01). When urinary concentrations of urea were expressed relative to creatinine, the relative concentration of urea remained low until a dietary concentration of 6.08 g SID Lys/kg, above which the relative concentration of urea increased (*P* < 0.01). Stillbirth rate increased linearly with increasing SID Lys concentration in the transition diet (*P* < 0.001), thus the concentration of SID Lys should be kept as low as possible without impairing sow performance excessively. A carry-over effect on milk yield was observed, showing that a dietary SID Lys concentration of 5.79 g/kg during transition optimized milk production at an average yield of 13.5 kg/d (*P* = 0.04). Increasing loss of body fat in lactation was observed with increasing SID Lys concentration in the transition diet (*P* = 0.03). In conclusion, the transition diet of multiparous sows should contain 5.79 g SID Lys/kg when fed 3.8 kg/d (13.0 MJ ME/kg), for a total SID Lys intake of 22 g/d.

## Teaser

This study demonstrates the importance of supplying dietary protein in accordance with the requirement of the multiparous sow in the transition period, to avoid detrimental effects of inadequate as well as excess protein supply. Milk yield was maximized at a dietary concentration of 5.79 g SID Lys/kg, while nitrogen utilization was optimized at 6.06 g SID Lys/kg when fed 3.8/d, however, stillbirth rate increased with increasing SID Lys concentration.

## Introduction

The transition period of the sow is often defined as the last week of gestation and the first three to five days into lactation and is characterized by profound physiological changes and rapid alterations in nutrient requirements (Theil et al., 2022). Fetal growth and mammary development are highly accelerated during transition (McPherson et al., 2004; Ji et al., 2006), and immunoglobulins are deposited in the mammary glands to be excreted in colostrum (Feyera et al., 2019). Factorial approaches show that the protein requirement of sows increases rapidly during the last week of gestation, while the energy requirement remains relatively stable (Feyera and Theil, 2017). A feed supply of 4.1 kg/d (12.9 MJ metabolizable energy [**ME**]/kg) during transition meets the requirements for optimal farrowing and subsequent lactational performance (Feyera et al., 2021; Bruun et al., 2023). Even though the optimal feed supply was established, the adequate concentration of dietary protein in these transition diets still needs to be determined. A recent study did not find improvements in mammary development in multiparous sows when feeding increased dietary Lys (14.8 vs. 20.8 g/d of standardized ileal digestible [**SID]** Lys) from days 90 to 110 of gestation (Farmer et al., 2023). Moreover, high dietary protein in lactation has been reported to impair energy utilization (Pedersen et al., 2019); thus, detrimental to the energy status of the sow (Feyera et al., 2018), and feeding more protein during gestation and lactation can reduce sow milk yield (**MY**) in organic sows (Johannsen et al., 2022). On the other hand, inadequate protein intake in late gestation impairs mammary development in gilts (Farmer et al., 2022) and can reduce lactation performance and cause mobilization of body protein (Kusina et al., 1999).

The objective of the present study was to determine the optimal concentration of dietary protein required for multiparous sows during the transition period, with a fixed dietary energy concentration and a feed supply of 3.8 kg/d, to ensure adequate dietary protein for efficient use of nutrients, a successful farrowing process and subsequent performance of sows and piglets.

## Material and Methods

The animal experimental procedures and care of animals were carried out in accordance with the Danish laws and regulations for the humane care and use of animals in research (Ministry of Food, Agriculture and Fisheries, The Danish Veterinary and Food Administration under act 474 of May 15, 2014, and executive order 2028 of December 14, 2020) and in compliance with the ARRIVE guidelines (Percie du Sert et al., 2020). A license to conduct the experiment was obtained from the Danish Animal Experiments Inspectorate (2018-15-0201-01484).

### Animals and housing

In total, 48 multiparous sows (DanBred Landrace × DanBred Yorkshire) were included in the experiment from day 108 of gestation until weaning on day 28 of lactation. The sows were inseminated with DanBred mixed Duroc semen. Sows were allotted to one of six dietary treatments according to parity and body weight (**BW**) on day 108 of gestation. The average parity in each treatment was 3.8 ± 0.2, with parities ranging from second to sixth. The experiment was conducted in two blocks, with 24 sows in each block.

Sows and piglets were housed individually in farrowing pens (2.7 × 1.7 m) on partially slatted flooring. Sows were crated throughout the entire period. Room temperature was kept at 22 °C. Lights were on from 0600 to 1800 hours and from 2230 to 2330 hours during the evening feeding. During the farrowings, light was continuously on. Until day 115 of gestation no bedding was supplied, after which straw and chopped straw were provided daily. A covered creep area was situated within each farrowing pen. Floor heating and infrared lamps were used to keep the ambient temperature at 32 °C in the creep area in which chopped straw was provided.

### Diets and feeding

The experiment was designed as a dose–response trial including five dietary treatments with increasing concentrations of crude protein (**CP**), here expressed as SID Lys. The ratio of other essential amino acids (**AA**) to Lys fulfilled or exceeded the Danish recommended AA composition for lactating sows (Tybirk et al., 2023). Two diets (low- and high-Lys diets) were formulated and mixed in different proportions to create the five experimental diets with increasing SID Lys concentrations (Table 1). A control treatment corresponding to a standard Danish lactation diet (Tybirk et al., 2023) was also included, as it is common practice to feed this diet in the last week of gestation in Denmark when sows have been moved to the farrowing section. From day 108 of gestation until 24 h after the onset of farrowing, sows were assigned to an experimental diet (*n* = 8 sows/treatment). The diets were mainly based on barley, wheat, and soybean meal (Table 2). In the low-Lys diet, soybean meal was substituted with wheat to reduce the concentration of SID Lys compared to the high-Lys diet. The low-Lys and high-Lys diets were formulated to contain 3.71 and 7.42 g SID Lys/kg as-fed, respectively (Table 3). The control diet was formulated to contain 8.56 g SID Lys/kg as-fed, which corresponds to the recommended concentration of Lys for lactating sows, considering a 5% margin of safety (Tybirk et al., 2023). The diets were formulated to be isoenergetic according to the Danish feed evaluation system, which is closely related to the net energy system (Patience, 2012). Using ME, the low-Lys, high-Lys, and control diets contained 12.9, 13.1, and 13.2 MJ/kg as-fed, respectively. The high-fiber ingredients, i.e., barley and sugar beet pulp, were kept at the same level in all diets. The indigestible dietary marker, titanium dioxide (**TiO**_**2**_), was added to the experimental diets (3.0 g/kg) to determine the apparent total tract digestibility (**ATTD**) of nitrogen (**N**). Sows were fed 3.8 kg/d corresponding to 49 to 50 MJ ME/d from day 108 of gestation until 24 h after the onset of farrowing. After this time, sows were fed the same standard lactation diet, formulated according to Danish recommendations. All sows were fed 3.8 kg/d on day 1 of lactation (day 0 being the day of farrowing) and progressively increasing to a maximum of 9.0 kg/d on day 17 of lactation. On days 7, 14, and 21 of lactation, the feed supply was reduced by 5% for every piglet that had died. Piglets were fed no supplementary feed during the lactation period.

**Table 1.** Proportions of the low-Lys, high-Lys, and control diet in the dietary treatments

	Dietary treatment					
	1	2	3	4	5	Control
Proportions, %						
Low-Lys	100	75	50	25	0	0
High-Lys	0	25	50	75	100	0
Control	0	0	0	0	0	100

**Table 2.** Ingredient composition of experimental diets

Ingredients, g/kg	Low-Lys	High-Lys	Control^1^
Barley	500	500	500
Wheat	418	287	251
Soybean meal, dehulled		124	158
Dried sugar beet pulp	40.0	40.0	40.0
Soy oil	9.43	16.0	17.8
Monocalcium phosphate	11.2	9.18	8.62
Calcium carbonate	13.3	13.2	13.2
Sodium chloride	3.89	3.89	3.89
L-Lys	1.86	2.84	3.26
DL-Met		0.57	0.94
L-Thr	0.33	1.04	1.31
L-Trp		0.005	0.08
L-Val		0.01	
Vitamin and mineral premix^2^	2.12	2.12	2.12
Vitamin E premix^3^	0.17	0.17	0.17

^1^The lactation diet had the same composition as the control diet.

^2^Pr. kg: 8,900 IU vitamin A, 890 IU vitamin D3, 159 mg vitamin E, 2.23 mg vitamin K3, 2.23 mg vitamin B1, 5.57 mg vitamin B2, 3.34 mg vitamin B6, 0.02 vitamin B12, 16.70 mg D-pantothenic acid, 22.26 niacin, 0.45 mg Biotin, 1.67 mg folic acid, 89.04 mg iron (FeSO_4_), 2.12 mg iodine, (Ca(IO_3_)_2_), 15.90 mg copper (CuSO_4_), 44.52 mg manganese (MnO), 106.0 mg Zink (ZnO), 0.32 mg selenium (Na_2_SeO_3_), 1,500 phytase activity units (Ronozyme HiPhos, dsm-firmenich Animal Nutrition & Health, Basel, Switzerland).

^3^Pr. kg: 50.000 IU vitamin E.

**Table 3.** Analyzed chemical composition of experimental diets

		Transition^1^		Lactation^2^
	Low-Lys	High-Lys	Control	
Chemical composition, g/kg				
DM	847	867	877	867
CP	87.0	130	145	151
SID CP^3^	69.5	109	122	127
Fat	35.0	40.0	43.0	39.0
Ash	42.0	46.0	48.5	47.0
Calcium	7.89	7.34	7.90	8.35
Phosphorous	5.01	4.81	4.95	5.33
Energy, MJ ME/kg^4^	12.9	13.1	13.2	13.2
Total amino acids, g/kg				
Lys	4.87	8.38	9.72	9.86
Met	1.45	2.54	3.08	3.05
Met + Cys	3.61	5.15	5.90	5.88
Thr	3.05	5.72	6.38	6.55
Ile	2.91	4.97	5.49	5.76
Leu	5.90	9.15	10.2	10.4
His	2.03	3.09	3.40	3.56
Phe	3.97	6.16	6.89	7.18
Phe + Tyr	6.49	10.3	11.5	11.8
Val	4.28	6.27	6.84	6.98
SID amino acids, g/kg^3^				
Lys	3.99	7.32	8.57	8.69
Met	1.20	2.27	2.81	2.78
Met + Cys	2.93	4.39	5.09	5.07
Thr	2.32	4.77	5.40	5.54
Ile	2.31	4.18	4.67	4.89
Leu	4.73	7.73	8.66	8.87
His	1.60	2.60	2.89	3.03
Phe	3.25	5.26	5.93	6.18
Phe + Tyr	5.21	8.66	9.77	10.0
Val	3.23	5.06	5.56	5.68

^1^Offered from day 108 of gestation until 24 h after the onset of farrowing.

^2^Offered from 24 h after the onset of farrowing until weaning on day 28 of lactation.

^3^SID: Standardized ileal digestible. The SID content of CP and amino acids was calculated from the analyzed content and corrected for the ratio between SID and total CP and amino acids content in the formulated diet based on Pedersen and Boisen (2002).

^4^The content of metabolizable energy (ME) was calculated using the EvaPig software (version 2.0.3.2; INRA, Saint-Gilles, France).

Sows were fed in three equal daily meals at 0700, 1500, and 2300 hours. The feed was provided by a pneumatic feeding system (SpotMix, Schauer Agrotronic, Prambachkirchen, Austria), which allowed for individual feeding and mixing of diets to obtain the designated dietary treatments. All diets were produced at Aarhus University, Campus Viborg’s feed factory. Leftovers were collected daily from 0900 to 1000 hours and were drained and weighed. To estimate the as-fed weight of wet leftovers, a regression line was obtained by soaking four samples (200, 500, 1,000, and 2,000 g) of each diet in water, followed by draining and weighing. Feed samples were collected during the experimental period and stored at −20 °C until analyzed.

### Recording and sampling

The BW and back fat thickness (**BF**) of sows were recorded on day 108 of gestation and days 2, 7, 14, 21, and 28 of lactation. The BF was measured using a digital ultrasound scanner (Lean-Meater, Renco Corp., Minneapolis, MN, USA) at P2, approximately 65 mm from either side of the spine at the last rib.

Blood samples were obtained from sows on day 108 of gestation, at the onset of farrowing, and on days 3, 10, 17, and 24 of lactation. Samples were collected 3 to 5 h after the morning meal, except for the onset of farrowing, where they were collected within an hour of birth of the first piglet. Sows were held using a snare restraint, and blood was collected from the jugular vein in a 9-mL Na-heparinized tube (Greiner BioOne GmBH, Kremsmünster, Austria) using a 18G 1.5’’ needle. At the onset of farrowing, blood was collected from the ear vein. On days 3 and 28 of lactation, sows were intramuscularly enriched with deuterium oxide (**D**_**2**_**O**; 0.0425 g 40% solution per kg BW) using a 21G 1.5’’ needle. Urine was used as background sample for the D_2_O analysis on day 3 of lactation. On day 28 of lactation, a blood sample was collected prior to D_2_O enrichment (background sample), and an additional blood sample was collected 4 to 6 h after the enrichment on both days (equilibrium sample). The blood samples were immediately centrifuged (1,558 × *g* at 4 °C), and the plasma was harvested into 2 mL microcentrifuge tubes. Plasma samples were stored at −20 °C until analyzed.

Colostrum samples were collected within 1 h and then 12, 24, and 36 h after the onset of farrowing. For these three last samples, sows were injected intramuscularly with 2 mL of oxytocin (10 IU/mL: Leopharma, Ballerup, Denmark) to induce colostrum letdown. The sows were manually milked from random teats while lying. Milk samples were also collected on days 3, 10, 17, and 24 of lactation after sows were injected with 0.3 mL of oxytocin in the ear vein. The sows were manually milked from random teats while standing. Teats were not emptied during milking. Piglets were not separated from the sow prior to sampling. Colostrum and milk samples were filtered through gauze into 50 mL falcon tubes and stored at −20 °C until analyzed.

Urine was collected on day 115 of gestation during a 6-h period and samples were pooled and weighed. Balloon catheters (size 20, Teleflex Medical, Puducherry, India) were inserted into the urinary bladder and a pin was applied to the catheter to prevent sliding. The catheters were emptied at least every second hour into a 10-L bottle and were closed immediately. The pooled urine was immediately analyzed for pH (B-71x LAQUAtwin, Horiba Scientific, NJ, USA) and subsamples were collected in 15 mL falcon tubes and 2 mL microcentrifuge tubes and stored at −20 °C until further analyzed. Spontaneous fecal samples were also collected during the urine collection, otherwise a grab sample was obtained after the urine collection. Fecal samples were stored immediately at −20 °C until analyzed.

The time of birth of each piglet was recorded and before suckling any colostrum the piglets were ear-tagged, and dried off, the umbilical cord was constricted and shortened to approximately 10 cm and birthweight was recorded. If birth intervals exceeded 1 h, farrowing assistance was applied. Blood samples were collected from every fourth piglet in the litter. The piglets were fixated in a cradle on their back and blood was drawn from the jugular vein in a 4-mL Na-heparinized tube (BD Vacutainer, Plymouth, UK) using a 22G 1’’ needle. Blood samples were immediately analyzed for blood gas metabolites (RAPIDPoint 500, Siemens Healthcare Diagnostics Ltd, UK). The BW of piglets were individually recorded at 12, 24, and 36 h after the onset of farrowing and on days 2, 7, 14, 21, and 28 of lactation. On day 2 of lactation, litters were equalized to at least 14 piglets or to the number of functional teats if more than 14. Male piglets were castrated on day 4 after birth. Piglets were locally anesthetized in the testicles (0.5 mL per testicle; Procamidor, 20 mg/mL; Salfarm Danmark A/S, Kolding, Denmark) and concomitantly intramuscularly injected with local analgesia (0.2 mL; Melovem, Dopharma B.V., VX Raamsdonksveer, The Netherlands). Dead piglets were collected and recorded continuously during the initial 24 h after farrowing, and daily for the remainder of lactation, to account for their intake of colostrum and milk, respectively, until death occurred.

### Chemical analysis

The chemical compositions (ash, crude fat, CP, calcium, phosphorus, and all AA except Trp) of the diets were analyzed in duplicates at a commercial laboratory (Eurofins Steins Laboratory A/S, Vejen, Denmark). The analyses were performed in accordance with European Commission Directives (Commission Regulation (EC), 2009). Diets were also analyzed for dry matter and TiO_2_. The dry matter content was determined by drying for 20 h at 103 °C in a forced air oven. The concentration of TiO_2_ was determined according to Short et al. (1996).

Plasma concentrations of glucose, lactate, triglycerides (**TG**), urea, and creatinine as well as urine concentrations of urea and creatinine were measured using spectrophotometry in accordance with standard procedures (Siemens Diagnostics Clinical Methods for ADVIA 1800). Plasma concentrations of nonesterified fatty acids (**NEFA**) were analyzed using the Wako, NEFA C ACS–ACOD assay method (Wako Chemicals GmbH, Neuss, Germany). All plasma samples were analyzed using an auto–analyzer (ADVIA 1800 Chemistry System, Siemens Medical Solutions, Terrytown, NY).

The composition (lactose, protein, and fat) of colostrum and milk was analyzed in triplicates using infrared spectroscopy (Milkoscan 4000, Foss, Hillerød, Denmark).

Fecal samples were analyzed for N, using the Dumas method (Hansen, 1989) on a Vario Max CN Element analyzer (Elementar Analysensytem GmbH, Langenselbold, Germany) and for dry matter and TiO_2_ as described for the transition diets. Urine samples were analyzed for N using the Kjeldahl method [method 984.13; AOAC (2000)] on a KjelTecTM 2400 (Foss, Hillerød, Denmark).

The atomic fraction of D_2_O in the injectate and plasma samples was measured as described by Theil et al. (2002b).

### Calculations and statistical analysis

Colostrum intakes (g/piglet) of individual piglets were estimated using the mechanistic model from Theil et al. (2014), with the following piglet characteristics as predictor variables: BW at birth (BWB; kg), 24 h BW gain (WG; g) and duration of suckling (D; min):


$$\begin{array}{llll} Colostrum\;intake= & \;-106+2.26\;WG+200\;BWB\; & +0.111\;D-1414\;WG/D\; & +0.0182\;WG/BWB.\;\;\; \end{array}$$

If piglets died within 24 h after the onset of farrowing, the time of death and BW at death were used to estimate the colostrum intake until death occurred. Colostrum yield (**CY**) was the sum of individual piglet colostrum intake within litter.

MY was estimated according to Hansen et al. (2012) using litter gain and litter size as predictors. The MY was estimated for each day from days 2 to 28 of lactation and the average weekly yield was calculated. If a sow was removed from the experiment before day 28 of lactation, the average MY for the last week was not calculated.

The gross energy of colostrum and milk was calculated using tabulated gross energy values for each colostrum and milk constituent measured [39.8 MJ/kg fat, 23.9 MJ/kg protein, and 16.5 MJ/kg lactose (Theil et al., 2020)].

The standardized ileal digestibility of CP and AA was calculated from the expected ratio of SID CP and AA contents to the total CP or AA content in the diet formulation based on Pedersen and Boisen (2002). The CP content of diets was calculated as the content of N multiplied by 6.25.

The ATTD of N was calculated using the concentration of N and TiO_2_ in the diets and feces, and the following formula:


$$\begin{array}{l} ATTD\;\;\;N,\;\%=100-\left(100\times \frac{{\left[TiO_{2}\right]}_{Diet}}{{\left[TiO_{2}\right]}_{Feces}}\times \frac{{\left[N\right]}_{Feces}}{{\left[N\right]}_{Diet}}\right) \end{array}$$

where ${\left[TiO_{2}\right]}_{Diet}$ and ${\left[TiO_{2}\right]}_{Feces}$ are the concentrations of TiO_2_ in the diets and feces, respectively, and $\;{\left[N\right]}_{Diet}$ and ${\left[N\right]}_{Feces}$ are the concentrations of N in the diets and feces, respectively.

The utilization of digested N was calculated as follows:


$$\begin{array}{l} Nutilization,\;\%=100\times \frac{Retained\;N\;\left(g/d\right)\;\;\;}{Digested\;N\;\left(g/d\right)} \end{array}$$

The D_2_O space was calculated in accordance with Theil et al. (2002b) using the D_2_O concentration in plasma before and after enrichment, and the mass and concentration of the D_2_O solution used for enrichment. Sow body protein and fat pools were estimated in accordance with Rozeboom et al. (1994) using the D_2_O space, BW, and BF.

The statistical analyses were performed with the software “R” (R Core Team, 2022), using the package “nlme” to analyze mixed models (Pinheiro et al., 2022), the package “lme4” to analyze mixed models with Poisson and negative binomial distributions (Bates et al., 2015), the package “car” to perform analysis of variance (ANOVA) using types II and III sum of squares (Fox and Weisberg, 2019), the package “emmeans” to obtain least squares means and orthogonal polynomial contrasts (Lenth, 2023) and the package “segmented” to perform breakpoint analysis (Muggeo, 2008).

All plasma metabolites and milk composition were analyzed using the following statistical mixed model with a Gaussian distribution:


$$\begin{array}{l} Y_{ijklm}=\mu +\alpha_{i}+\beta_{j}+\gamma_{k}+{\left(\alpha \beta \right)}_{ij}+\nu_{l}+\tau_{m}+\varepsilon_{ijklm} \end{array}$$

where $Y_{ijklm}\;$ is the observed trait, μ is the overall mean, $\alpha_{i}$ is the effect of dietary treatment (i = 1, 2, 3, 4, 5 or control), $\beta_{j}$ is the effect of day in gestation or lactation (*j* = day 108 of gestation, onset of farrowing, days 3, 10, 17, or 24 of lactation), $\gamma_{k}$ is the effect of parity of sows (*k* = 2 to 3 or 4 to 6), ${\left(\alpha \beta \right)}_{ij}$ is the interaction between dietary treatment and day in gestation or lactation, $\nu_{l}\;$ is the random effect of sow (*l* = 1, 2,…, 48), $\tau_{m}$ is the random effect of block (*m* = 1 or 2) and $\varepsilon_{ijklm}$ is the residual random components. Colostrum composition were analyzed using a similar model for 0, 12, 24, and 36 h after onset of farrowing. The MY of the sows and litter average daily gain (**ADG**) were analyzed using a similar model but on a weekly basis (*j* = weeks 1, 2, 3, and 4 of lactation).

Sow performance, feed and nutrient intake, sow body compositions, litter characteristics, CY, urine metabolites, and N balance were analyzed using the following statistical mixed model with a Gaussian distribution:


$$\begin{array}{l} Y_{ijk}=\mu +\alpha_{i}+\beta_{j}+\nu_{k}+\varepsilon_{ijk} \end{array}$$

where $Y_{ijk}\;$ is the observed trait, μ is the overall mean, $\alpha_{i}$ is the effect of dietary treatment (*i* = 1, 2, 3, 4, 5, or control), $\beta_{j}$ is the effect of parity of sows (*j* = 2 to 3 or 4 to 6), $\nu_{l}\;$ is the random effect of block (*k* = 1 or 2) and $\varepsilon_{ijk}$ is the residual random components. In the model for BW and BF change from days 2 to 28 of lactation, the BW and BF on day 2 of lactation, respectively, were included as a covariates. Also, in the models for body protein and fat pool on day 3 of lactation, sow BW on day 2 was included as a covariate. Piglet performance and birth interval were analyzed using a similar model but with the random effect of sow. In the model for piglet ADG, the BW on day 2 and in the model for litter ADG, the LW at day 2 was included as covariate. The stillbirth rate was analyzed using a similar model with a Poisson distribution, and total-born piglets was included as an offset. Also, the farrowing assistance rate and birth interval were analyzed using a similar model but with a negative binomial distribution.

Orthogonal polynomial contrasts were used to evaluate the linear and quadratic effects of dietary SID Lys concentration. Broken-linear models were fitted to variables that showed linear and/or quadratic effects in order to estimate breakpoints for these variables, using the actual concentration of SID Lys/kg in the diet for each sow. Linear models were fitted if no breakpoint was detected. Significant or tendencies of broken-linear and linear responses are presented in their respective tables.

Results on normally distributed data are presented as least squares means with the greatest SEM values. If an interaction was not significant, it was removed from the model and not included in the final analysis. Results on log-transformed data are presented as the back-transformed least squares means and 95% confidence intervals. Results were considered significant at *P* < 0.05 and tendencies when *P* < 0.10. The *P* values reported for the linear and broken-linear models are the *P* values for slope.

## Results

Three sows were excluded from the experiment: one refused to eat during transition, another died from splenic torsion during lactation, and the third exhibited serious welfare concerns during lactation.

### Sow feed intake and body composition

The average daily intake of SID Lys during transition increased linearly with increased SID Lys inclusion in the dietary treatments (Table 4; *P *< 0.001), whereas the ADFI during transition was not affected by dietary treatment and averaged 3.7 kg/d. The ADFI during lactation showed a quadratic response to dietary concentration of SID Lys during transition (*P* < 0.01) and a tendency for a broken-linear relationship with SID Lys concentration during transition (*P *= 0.05), with a breakpoint at 4.91 g SID Lys/kg in the transition diet; at which time ADFI reached a plateau of 7.2 kg/d.

**Table 4.** Effect of increasing dietary SID Lys during transition on sow body composition, feed intake and litter performance

	Dietary treatment^1^, SID Lys g/kg							Parity			*P* value^2^			
Item^3^^,^^4^	3.99	4.79	5.61	6.45	7.32	Control (8.57)	SEM	2 to 3	4 to 6	SEM	Trt	Parity	Lin	Quad
*n* (at farrowing)	8	7	8	8	8	8		20	27					
Sows														
Parity	2.8	2.8	2.6	2.9	2.8	2.9	0.4				0.99		0.90	0.91
Sow BW (day 108), kg	289	291	289	288	291	293	9.3	285	296	5.9	1.00	0.17	0.95	0.92
Sow BW (day 2), kg	274	267	269	274	273	281	9.1	267	279	5.4	0.91	0.09	0.83	0.59
Sow BW change (days 2 to 28), kg	-4.8	-5.7	-11.0	-11.0	-9.7	-14.2	3.6	-12.0	-6.8	2.1	0.33	0.06	0.12	0.48
Sow BF (day 108), mm	13.6	11.7	13.1	14.0	12.3	12.7	1.0	12.2	13.5	0.6	0.49	0.09	0.49	0.51
Sow BF (day 2), mm	14.0	12.1	12.8	14.0	11.6	13.5	1.0	12.4	13.6	0.7	0.47	0.16	0.30	0.87
Sow BF change (days 2 to 28), mm	-3.1	-3.2	-2.6	-3.0	-2.0	-2.3	0.7	-2.7	-2.7	0.5	0.36	0.99	0.12	0.50
Body protein pool (day 3), kg	43.9	44.2	44.0	43.6	44.3	44.1	0.39	44.1	44.0	0.24	0.73	0.69	0.87	0.73
Body protein change (days 3 to 28), kg	-2.9	-3.4	-2.6	-2.6	-2.4	-3.2	0.6	-3.0	-2.7	0.5	0.55	0.34	0.16	0.70
Body fat pool (day 3), kg	68.2	66.9	67.6	69.9	66.4	67.2	2.4	67.3	68.2	1.5	0.86	0.64	0.92	0.80
Body fat change (days 3 to 28), kg	-3.78	-7.37	-9.38	-10.4	-8.66	-11.0	2.29	-10.2	-6.68	1.30	0.23	0.048	0.07	0.15
Feed intake														
ADFI (transition), kg/d	3.60	3.69	3.69	3.68	3.74	3.66	0.06	3.67	3.68	0.04	0.61	0.81	0.07	0.73
SID Lys intake (transition), g/d	14.3^f^	17.7^e^	21.0^d^	23.7^c^	27.4^b^	31.4^a^	0.4	22.6	22.6	0.3	<0.001	0.81	<0.001	0.84
ADFI (lactation), kg/d^3^	6.76^b^	7.16^ab^	7.26^ab^	7.30^ab^	7.01^ab^	7.37^a^	0.14	7.15	7.14	0.08	0.02	0.91	0.16	<0.01
Litter														
Total born	19.1^b^	23.4^ab^	24.9^ab^	22.5^ab^	25.9^a^	25.3^a^	1.5	23.5	23.5	0.9	0.03	0.78	<0.01	0.25
Liveborn	18.1	21.9	22.6	19.5	20.6	21.1	1.5	20.6	20.7	0.9	0.28	0.97	0.56	0.09
Stillbirth rate, %	4.9^b^ [2.4 to 10.1]	6.8^ab^ [3.3 to 14.0]	9.3^ab^ [4.5 to 19.1]	13.3^ab^ [6.5 to 27.4]	15.3^a^ [7.4 to 31.5]	15.2^a^ [7.4 to 31.4]		10.1 [6.3 to 16.3]	9.7 [6.0 to 15.7]		<0.01	0.84	<0.001	0.77
Farrowing assistance rate, %	16.2 [10.3 to 25.6]	11.4 [7.2 to 18.0]	10.1 [6.4 to 16.0]	6.4 [4.0 to 10.1]	12.0 [7.6 to 19.0]	3.3 [2.1 to 5.2]		9.8 [6.7 to 14.5]	7.9 [5.3 to 11.6]		0.52	0.67	0.20	0.31
Farrowing duration, h	6.56	7.75	6.54	6.66	7.71	6.44	1.10	7.54	6.35	0.74	0.86	0.16	0.72	0.81
Litter weight (birth), kg	24.3	27.5	28.7	25.4	26.6	25.8	2.0	27.2	25.5	1.2	0.65	0.26	0.71	0.26
Colostrum yield (24 h), kg	6.65	6.87	7.46	7.13	7.11	7.15	0.54	7.20	6.92	0.31	0.90	0.49	0.48	0.49

Response	Model^5^		Breakpoint		Equation^6^							*P* value (slope)		
Body fat change (days 3 to 28), kg^7^	L		NA		Y_*i*_ = –2.81_Parities 2 to 3_ or 0.77_Parities 4 to__6_ – 1.22 × *X*_*i*_							0.03		
ADFI (lactation), kg/d	BL		4.91		Y_*i*_ = 7.13 – 0.50 × (4.91—*X*_*i*_) for *X*_*i*_ < 4.91							0.05		
Stillbirth rate, %	L		NA		Y_*i*_ = exp(–3.68 + 0.23 × *X*_*i*_)							<0.001		

^a,b,c,d,e,f^Within a row, values without common subscriptions differ (*P < *0.05).

^1^Dietary treatments were fed from day 108 of gestation until 24 h after the onset of farrowing.

^2^Orthogonal contrasts were used to evaluate linear (Lin) and quadratic (Quad) effects of dietary SID Lys.

^3^BW: Body weight, BF: Back fat, ADFI: Average daily feed intake.

^4^Sow body protein and fat pools were estimated using the D_2_O technique and prediction equations of Rozeboom et al. (1994).

^5^L: linear relationship, BL: linear-broken relationship.

^6^Y_*i*_ being the response variable and *X*_*i*_ being the dietary concentration of SID Lys (g/kg) during transition.

^7^Effect of parity (*P *= 0.04).

Sow BW and BF were not affected by the dietary treatment during the transition and lactation periods (Table 4). Sow BW on day 2 of lactation and BF on day 108 of gestation tended to be greater in parities 4 to 6 than in parities 2 to 3 sows (*P* = 0.09). Younger sows also tended to have a greater BW loss during lactation compared to older sows (*P* = 0.06). Sows’ body fat change from days 3 to 28 of lactation showed a negative linear relationship with SID Lys during the transition period, indicating higher body fat losses at increasing SID Lys concentration (Table 4, *P* = 0.03).

### Farrowing performance and colostrum

**Figure 1.**
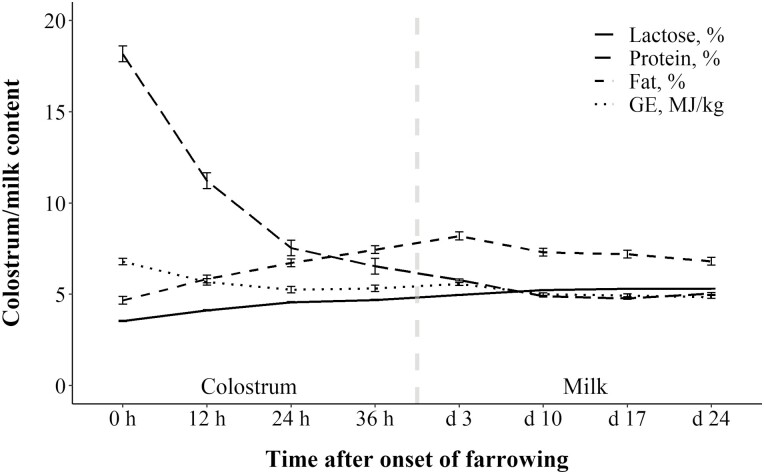
The effect of time after onset of farrowing on colostrum and milk macro nutrient composition and gross energy content from sows fed increasing concentrations of SID Lys during the last week of gestation (*P* < 0.001 for all). Error bars indicate SEM. The gray vertical line indicates the change from colostrum to milk.

The stillbirth rate was affected by dietary treatment (Table 4, *P* = 0.01) and the orthogonal contrast revealed a linear relationship between stillbirth rate and SID Lys during transition (*P* < 0.001): Dietary concentration of SID Lys had a coefficient of 0.23 (*P* < 0.001), hence for every unit increase in SID Lys concentration (g/kg feed), stillbirth rate increased by 25%. The number of total-born piglets was affected by dietary treatment (*P *= 0.03) and showed a positive linear relationship with SID Lys during transition (*P* < 0.01). There were no dietary treatment effects on the number of liveborn piglets, farrowing duration, rate of farrowing assistance, or litter weight at birth. The CY was not affected by dietary treatment and averaged 7.0 kg within the first 24 h after the onset of farrowing (Table 4). Colostrum composition was not affected by dietary treatment, but all measured components were affected by time after onset of farrowing (*P* < 0.001, Fig. 1).

### Litter and piglet performance

**Figure 2.**
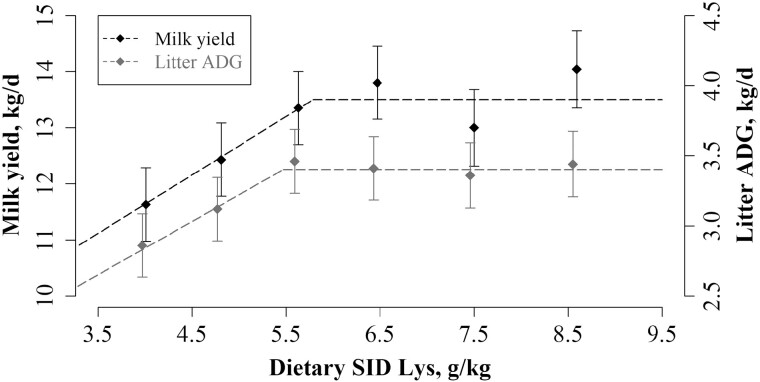
The effect of increasing concentration of SID Lys in the last week of gestation on sow MY and litter ADG. Data was best described by a broken-linear model and the breakpoint is presented together with SEM and the *P* value for the slope below the breakpoint is presented. The MY increased until a breakpoint at 5.79 ± 0.63 g/kg of SID Lys and reached a plateau at 13.5 kg/d (*P* = 0.04). For *X*_*i*_ < 5.79, MY, kg/d = 13.5 – 1.04 × (5.79 – *X*_*i*_), where *X*_*i*_ is the concentration of SID Lys, g/kg, for the individual sow, *i*. The litter ADG increased until a breakpoint at 5.47 ± 0.76 g/kg of SID Lys and reached a plateau at 3.40 kg/d (*P* = 0.16). For *X*_*i*_ < 5.47, litter ADG, kg/d = 3.40 – 0.38 × (5.47 – *X*_*i*_), where *X*_*i*_ is the concentration of SID Lys, g/kg, for the individual sow, *i*. The diamonds (♦) represent the least squares means and the error bars indicate SEM for the least squares mean.

Neither piglet BW at birth and at 24 h after the onset of farrowing nor birth interval were affected by dietary treatment (Table 5). At weaning litter size tended to show a quadratic response to dietary treatment (*P* = 0.08) and the broken-linear relationship showed a tendency to a linear increase in litter size until 4.95 g SID Lys/kg (*P* = 0.10), after which there was a plateau of 12.2 piglets per litter. Litter weight at weaning tended to show linear and quadratic responses to dietary treatment (*P *= 0.06 and *P* = 0.10, respectively), and a broken-linear relationship showed that litter weight at weaning tended to peak at 5.66 g SID Lys/kg at 109 kg per weaned litter (*P* = 0.06). No differences in piglet BW at weaning between dietary treatments were found (Table 5, *P *= 0.49). All analyzed components of milk were affected by time after onset of farrowing (Fig. 1). There was a linear response (*P* = 0.03) and a tendency of a quadratic response (*P* = 0.10) between MY and dietary treatment. The broken-linear relationship showed that MY was maximized (13.5 kg/d) when sows were fed 5.79 g SID Lys/kg during transition (Fig. 2; *P* = 0.04). Similarly, a linear response (*P* = 0.01) and a tendency of a quadratic response (*P* = 0.08) were found between litter ADG and dietary treatment. The broken-linear relationship was not significant but indicated that litter ADG was maximized at 3.40 kg/d, when sows were fed 5.47 g SID Lys/kg during transition (Fig. 2; *P* = 0.16).

**Table 5.** Effect of increasing dietary SID Lys during transition on piglet performance

	Dietary treatment^1^, SID Lys g/kg							Parity			*P* value^2^			
Item^3^	3.99	4.79	5.61	6.45	7.32	Control (8.57)	SEM	2 to	4 to 6	SEM	Trt	Parity	Lin	Quad
*n* (at birth)	141	158	147	151	159	164		372	548					
Birth interval, min	21.4 [15.6 to 29.5]	16.9 [12.2 to 23.3]	16.4 [11.9 to 22.7]	18.9 [13.7 to 26.1]	17.7 [12.9 to 24.5]	17.9 [13.0 to 24.6]		19.3 [15.5 to 24.1]	17.1 [13.7 to 21.3]		0.85	0.38	0.17	0.15
Piglet body weight (birth), kg	1.36	1.26	1.27	1.33	1.27	1.25	0.06	1.33	1.25	0.04	0.79	0.09	0.41	0.08
Piglet body weight (24 h), kg	1.43	1.32	1.34	1.40	1.35	1.32	0.07	1.41	1.32	0.04	0.76	0.09	0.49	<0.01
Piglet body weight (day 2), kg	1.68	1.57	1.63	1.68	1.57	1.57	0.08	1.66	1.57	0.05	0.72	0.14	0.29	1.00
Piglet body weight (day 28), kg	9.03	8.40	9.14	8.92	8.90	8.64	0.46	9.03	8.65	0.37	0.49	0.12	0.28	0.80
Piglet ADG (days 2 to 28), kg/d	0.217	0.225	0.251	0.218	0.236	0.232	0.02	0.235	0.225	0.01	0.18	0.30	0.43	0.37
Litter size (day 28)	10.7	12.0	12.0	12.4	11.8	12.5	0.6	11.9	11.8	0.3	0.22	0.86	0.12	0.08
Litter weight (day 28), kg	93.2	100.7	109.4	110.3	104.7	112.6	7.5	107.0	103.4	5.9	0.11	0.42	0.06	0.10

Response	Model^4^		Breakpoint		Equation^5^							*P* value (slope)		
Litter size (day 28)	BL		4.95		Y_*i*_ = 12.2 – 1.54 × (4.95 – *X*_*i*_) for *X*_*i*_ < 4.95							0.10		
Litter weight (day 28), kg	BL		5.66		Y_*i*_ = 109 – 9.62 × (5.66 – *X*_*i*_) for *X*_*i*_ < 5.66							0.06		

^a,b^Within a row, values without common subscriptions differ (*P < *0.05).

^1^Dietary treatments were fed from day 108 of gestation until 24 h after the onset of farrowing.

^2^Orthogonal contrasts were used to evaluate linear (Lin) and quadratic (Quad) effects of dietary SID Lys.

^3^ADG: Average daily gain.

^4^L: linear relationship, BL: linear-broken relationship.

^5^Y_*i*_ being the response variable and *X*_*i*_ being the dietary concentration of SID Lys (g/kg) during transition.

No dietary treatment effects on piglets’ blood biochemical parameters at birth were found (Table 6). Sow parity affected the concentrations of lactate, O_2_, CO_2_, and HCO_3_ in piglets. Concentrations of lactate (*P *= 0.03) and O_2_ (*P *= 0.02) were lower, while CO_2_ (*P *= 0.02) and HCO_3_ (*P *= 0.03) were greater in piglets from second to third parity sows compared with piglets from forth to sixth parity sows.

**Table 6.** Effect of increasing dietary SID Lys during transition on piglet blood biochemical parameters at birth.

	Dietary treatment^1^, SID Lys g/kg							Parity			*P*-value^2^			
Item	3.99	4.79	5.61	6.45	7.32	Control (8.57)	SEM	2 to 3	4 to 6	SEM	Trt	Parity	Lin	Quad
*n*	24	25	33	28	37	28		68	107					
pH	7.37	7.35	7.38	7.34	7.36	7.35	0.03	7.34	7.37	0.02	0.84	0.26	0.30	1.00
Glucose	2.69	2.43	2.51	2.62	2.46	2.82	0.22	2.59	2.58	0.13	0.75	0.99	0.68	0.87
Lactate	4.89	5.81	5.53	5.99	6.30	6.32	0.50	5.37	6.24	0.30	0.25	0.03	0.04	0.79
O_2_	5.35	4.96	5.14	5.34	5.15	5.63	0.41	4.92	5.60	0.30	0.78	0.02	0.98	0.67
CO_2_	29.1	27.5	28.6	28.4	28.0	26.7	1.2	29.1	27.0	0.7	0.71	0.02	0.71	0.75
HCO_3_	27.6	26.0	27.2	26.8	26.5	25.3	1.1	27.5	25.6	0.7	0.70	0.03	0.70	0.75
Anion gap	11.9	12.6	12.0	13.2	13.6	13.8	1.0	12.4	13.4	0.6	0.57	0.21	0.15	0.66

^a,b^Within a row, values without common subscriptions differ (*P < *0.05).

^1^Dietary treatments were fed from day 108 of gestation until 24 h after the onset of farrowing.

^2^Orthogonal contrasts were used to evaluate linear (Lin) and quadratic (Quad) effects of dietary SID Lys.

### Sow plasma and urine metabolites

Plasma NEFA concentrations were greater when sows were fed 3.99 g SID Lys/kg compared with sows fed 7.32 g SID Lys/kg (Table 7; *P* = 0.02). There was a tendency for a negative linear effect of dietary SID Lys on NEFA concentrations (*P* = 0.08), with a 3.5% decrease in plasma NEFA for every unit increase in SID Lys concentration (g/kg feed). There was a dietary treatment by time interaction on plasma creatinine, with lower plasma creatinine being present at the onset of farrowing in sows fed the control vs. treatment diets (*P *= 0.03). Plasma creatinine concentration was greater in sows of parities 4 to 6 compared to parities 2 to 3 (*P* = 0.04). All plasma metabolites, except lactate, were affected by time (*P* < 0.001 for all).

**Table 7.** Effect of increasing dietary SID Lys during transition on sow plasma metabolites on day 108 of gestation, onset of farrowing, and days 3, 10, 17, and 24 of lactation

	Dietary treatment^1^, SID Lys g/kg							Parity			*P*-value^2^	
Item^3^	3.99	4.79	5.61	6.45	7.32	Control (8.57)	SEM	2 to 3	4 to 6	SEM	Trt	Parity
Glucose, mM	5.30	5.40	5.24	5.44	5.08	5.35	0.11	5.32	5.28	0.07	0.19	0.59
Lactate, mM	2.06 [0.57 to 1.69]	1.76 [0.34 to 1.3]	1.79 [0.37 to 1.34]	1.74 [0.33 to 1.27]	2.09 [0.6 to 1.74]	1.84 [0.41 to 1.41]		1.86 [0.48 to 1.34]	1.89 (0.5 to 1.38)		0.44	0.76
TG, mM	0.21	0.22	0.19	0.24	0.19	0.21	0.03	0.22	0.20	0.02	0.60	0.35
NEFA, µeq/L	139^a^ [75.4 to 251]	107^ab^ [57.8 to 193]	112^ab^ [60.4 to 202]	121^ab^ [65.7 to 219]	98.1^b^ [53 to 177]	114^ab^ [61.9 to 207]		122 [66.9 to 218]	108 (58.9 to 192)		0.02	0.05
Urea, mM	3.14	3.32	3.38	3.40	3.24	3.39	0.33	3.22	3.40	0.31	0.84	0.21
Creatinine, µM	163	174	170	176	162	160	8.4	161	174	5.30	0.62	0.04

	Time							*P* value^2^				
	Day 108	Onset	Day 3	Day 10	Day 17	Day 24	SEM	Time	Trt × Time	Lin	Quad	
Glucose, mM	4.72^c^	5.60^a^	5.08^b^	5.51^a^	5.45^a^	5.44^a^	0.09	<0.001	0.10	0.27	0.20	
Lactate, mM	2.03 [0.6 to 1.56]	1.75 [0.39 to 1.21]	2.00 [0.58 to 1.52]	1.84 [0.46 to 1.32]	1.85 [0.46 to 1.33]	1.80 [0.43 to 1.27]		0.30	0.53	0.99	0.048	
TG, mM	0.57^a^	0.21^b^	0.21^b^	0.10^c^	0.08^c^	0.08^c^	0.02	<0.001	0.44	0.87	0.57	
NEFA, µeq/L	76.8^c^ [41.3 to 138]	379^a^ [208 to 687]	106^b^ [57.4 to 191]	109^b^ [59.1 to 197]	87.1^bc^ [47.0 to 157]	76.5^c^ [41.2 to 138]		<0.001	0.11	0.02	0.60	
Urea, mM	3.22^cd^	2.37^e^	2.90^d^	3.47^bc^	3.74^b^	4.16^a^	0.31	<0.001	0.19	0.60	0.26	
Creatinine, µM	185^b^	212^a^	159^c^	151^c^	148^c^	149^c^	4.6	<0.001	0.03	0.95	0.21	

Response	Model^4^		Breakpoint		Equation^5^					*P* value (slope)		
NEFA, µeq/L^6^	L		NA		Y_*i*_ = exp(β_1_ – β_2_ – 0.035 × *X*_*i*_)					0.08		

^a,b,c,d,e^Within a row, values without common subscriptions differ (*P < *0.05).

^1^Dietary treatments were fed from day 108 of gestation until 24 h after the onset of farrowing.

^2^Orthogonal contrasts were used to evaluate linear (Lin) and quadratic (Quad) effects of dietary SID Lys.

^3^TG: triglycerides, NEFA: nonesterified fatty acids.

^4^L: linear relationship, BL: linear-broken relationship.

^5^Y_*i*_ being the response variable and *X*_*i*_ being the dietary concentration of SID Lys (g/kg) during transition.

^6^Effect of time (*P* < 0.001): β_1_ = 4.61, 6.21, 4.93. 4.96, 4.73 and 4.60 for day 108 of gestation, onset of farrowing, day 3, 10, 17 and 24 of lactation, respectively, and tendency on effect of parity (*P* = 0.08): β_2_ = 0 and 0.11 for parities 2 to 3 and 4 to 6, respectively.

**Figure 3.**
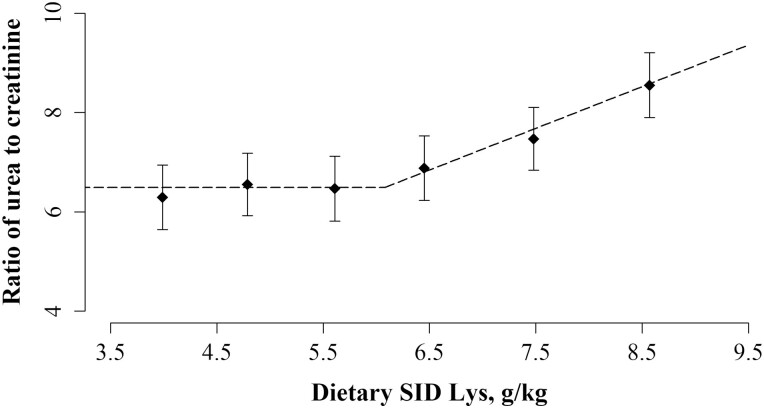
The effect of increasing concentration of SID Lys in the last week of gestation on the ratio of urea to creatinine in urine at day 115 of gestation. Data was best described by a broken-linear model and the breakpoint is presented together with SEM and the *P* value for the slope above the breakpoint is presented. The ratio of urea to creatinine remained stable until a breakpoint at 6.08 ± 0.58 g/kg of SID Lys and increased after the breakpoint (*P* < 0.01). For *X*_*i*_ > 6.08, ratio of urea to creatinine = 6.49 + 0.84 × (*X*_*i*_ – 6.08). The value, *X*_*i*_, is the concentration of SID Lys, g/kg, for the individual sow, *i*. The black diamonds (♦) represent the least squares means and the error bars indicate SEM for the least squares mean.

The pH of sow urine on day 115 of gestation showed a positive linear relationship with dietary SID Lys during transition (Table 8; *P* < 0.001). A broken-linear relationship was identified between the ratio of urea to creatinine and dietary SID Lys (*P* < 0.01), the ratio of urea to creatinine remained stable until 6.08 g SID Lys/kg, after which it increased linearly (Fig. 3). Dietary treatment did not affect the daily urine production or excretion of urea and creatinine. There was a tendency to a dietary treatment by time interaction on plasma glucose, however, not with an interpretable pattern.

**Table 8.** Effect of increasing dietary SID Lys during transition on sow urine production, urinary pH, urea, and creatinine on day 115 of gestation

	Dietary treatment^1^, SID Lys g/kg							Parity			*P*-value^2^			
Item	3.99	4.79	5.61	6.45	7.32	Control (8.57)	SEM	2 to 3	4 to 6	SEM	Trt	Parity	Lin	Quad
Urine production, kg/d	9.8 [3.0 to 23.1]	12.1 [3.9 to 28.8]	11.0 [3.4 to 26.0]	9.2 [2.7 to 21.7]	8.7 [2.5 to 20.4]	16.6 [5.7 to 39.9]		10.5 [3.8 to 21.7]	11.4 [4.3 to 23.8]		0.62	0.72	0.55	0.53
pH	6.2^bc^	6.1^c^	6.4^abc^	6.7^ab^	6.6^abc^	6.9^a^	0.16	6.4	6.5	0.12	<0.001	0.25	<0.01	0.91
Urea, mmol/d	500	549	540	502	533	652	65	514	578	51	0.30	0.10	0.91	0.55
Creatinine, mmol/d	81.1	83.1	84.4	73.2	73.2	77.0	6.1	76.9	80.5	3.7	0.61	0.46	0.15	0.41
Urea:Creatinine	6.29^b^	6.55^b^	6.47^b^	6.88^ab^	7.47^ab^	8.55^a^	0.64	6.81	7.26	0.54	0.02	0.71	0.02	0.42

Response	Model^3^		Breakpoint		Equation^4^								*P* value (slope)	
pH	L		NA		Y_*i*_ = 5.53 + 0.16 × *X*_*i*_								<0.001	
Urea:Creatinine	BL		6.08		Y_*i*_ = 6.49 + 0.84 × (*X*_*i*_ – 6.08) for *X*_*i*_ > 6.08								<0.01	

^a,b,c^Within a row, values without common subscriptions differ (*P < *0.05).

^1^Dietary treatments were fed from day 108 of gestation until 24 h after the onset of farrowing.

^2^Orthogonal contrasts were used to evaluate linear (Lin) and quadratic (Quad) effects of dietary SID Lys.

^3^L: linear relationship, BL: linear-broken relationship.

^4^Y_*i*_ being the response variable and *X*_*i*_ being the dietary concentration of SID Lys (g/kg) during transition.

### Sow nitrogen balance

**Figure 4.**
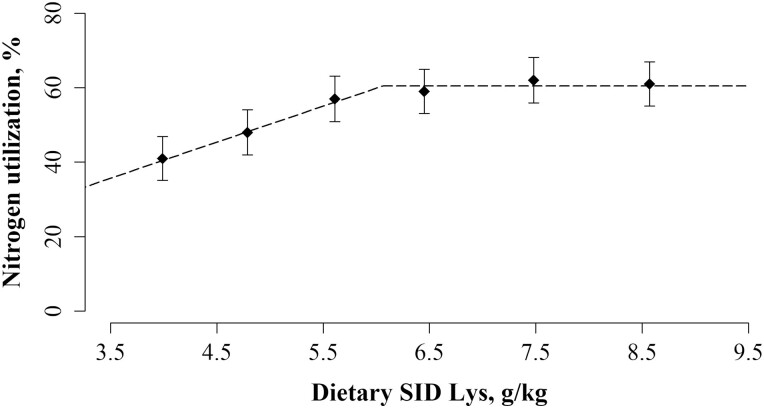
The effect of increasing concentration of SID Lys in the last week of gestation on nitrogen (N) utilization at day 115 of gestation. Data was best described by a broken-linear model and the breakpoint is presented together with SEM and the P value for the slope below the breakpoint is presented. The N utilization increased until a breakpoint at 6.06 ± 0.52 g/kg of SID Lys and reached a plateau at 60.5% (*P* < 0.01). For *X*_*i*_ < 6.06, N utilization, % = 60.5 – 9.7 * (6.06 – *X*_*i*_). The value, *X*_*i*_, is the concentration of SID Lys, g/kg, for the individual sow, *i*. The black diamonds (♦) represent the least squares means and the error bars indicate SEM for the least squares mean.

In Table 9, the effects of dietary treatment on ATTD and sow N balance on day 115 of gestation are presented. All variables, except urine N output, were affected by dietary treatment (*P* < 0.001). The ATTD of N, realized N intake, fecal N output, retained N, and N utilization increased linearly with dietary SID Lys (*P* < 0.001 for all). A broken-linear relationship was present between N utilization and dietary SID Lys concentration, with the N utilization increasing until 6.06 SID Lys/kg and reaching a plateau at 60.5% (Fig. 4).

**Table 9.** Effect of increasing dietary SID Lys during transition on ATTD and sow nitrogen (N) balance on day 115 of gestation

	Dietary treatment^1^, SID Lys g/kg							Parity			*P*-value^2^			
Item	3.99	4.79	5.61	6.45	7.32	Control (8.57)	SEM	2 to 3	4 to 6	SEM	Trt	Parity	Lin	Quad
ATTD of N, %	74.0^c^	75.5^bc^	78.3^ab^	78.3^ab^	79.1^a^	80.4^a^	0.8	77.6	77.5	0.5	<0.001	0.85	<0.001	0.13
Realized N intake, g/d	50.0^f^	57.7^e^	63.9^d^	69.9^c^	77.8^b^	86.9^a^	0.9	67.3	68.1	0.6	<0.001	0.25	<0.001	0.93
Fecal N output, g/d	12.3^c^	13.5^bc^	13.5^bc^	14.8^ab^	16.2^a^	16.6^a^	0.7	14.5	14.5	0.4	<0.001	0.98	<0.001	0.32
Urine N output, g/d	21.5	22.5	21.6	21.6	23.2	26.3	2.9	21.2	24.4	2.2	0.63	0.08	0.66	0.81
Retained N, g/d	15.6^d^	21.0^cd^	28.5^bc^	31.8^ab^	38.4^a^	38.5^a^	3.3	30.4	27.5	2.7	<0.001	0.12	<0.001	0.84
N utilization, %	41.1^b^	48.2^ab^	56.8^ab^	58.9^a^	62.4^a^	61.4^a^	6.1	57.6	52.0	5.1	<0.001	0.11	<0.001	0.37
Response	Model^3^		Breakpoint	Equation^4^									*P* value (slope)	
ATTD of N, %	L		NA	Y_*i*_ = 69.2 + 1.37 × *X*_*i*_									<0.001	
Realized N intake, g/d	L		NA	Y_*i*_ = 17.8 + 8.06 × *X*_*i*_									<0.001	
Fecal N output, g/d	L		NA	Y_*i*_ = 9.69 + 0.87 × *X*_*i*_									<0.001	
Retained N, g/d	L		NA	Y_*i*_ = -4.84 + 5.51 × *X*_*i*_									<0.001	
N utilization, %^5^	BL		6.06	Y_*i*_ = 50.6_Parities 2 to 3_ or 45.6_Parities 4 to 6_ – 0.08 × (6.06 – *X*_*i*_) for *X*_*i*_ < 6.06									<0.01	

^a,b,c,d,e,f^Within a row, values without common subscriptions differ (*P < *0.05).

^1^Dietary treatments were fed from day 108 of gestation until 24 h after the onset of farrowing.

^2^Orthogonal contrasts were used to evaluate linear (Lin) and quadratic (Quad) effects of dietary SID Lys.

^3^L: linear relationship, BL: linear-broken relationship.

^4^Y_*i*_ being the response variable and *X*_*i*_ being the dietary concentration of SID Lys (g/kg) during transition.

^5^Tendency on effect of parity (*P* = 0.09).

## Discussion

Experimental diets were composed using the Danish recommended AA composition for lactating sows (Tybirk et al., 2023). Assuming this AA composition resembles the sow’s actual requirement, dietary treatment effects were attributable to the overall supply of protein and not to any single essential AA. However, it should be kept in mind that of the nine essential AA, Lys has been studied most extensively in gestating sows (Samuel et al., 2012; Ramirez-Camba et al., 2020; Thomas et al., 2021; Farmer et al., 2022, 2023), while only a few studies have been conducted on the other essential AA [methionine; Bin et al. (2018); Xia et al. (2019), threonine; Dourmad and Étienne (2002); Levesque et al. (2011), tryptophan; Franco et al. (2014) and isoleucine; Franco et al. (2013)]. According to Liebig’s law of minimum, the essential AA fed with the most deficit is the limiting factor for AA utilization. Lys is often the first limiting AA in diets for pigs, thus the protein supply was expressed as SID Lys in the present study.

### Impact of dietary protein on nitrogen balance

The broken-linear relationship showed that N utilization increased until 6.06 g SID Lys/kg with a maximum N utilization of 60.5% during the transition period. At dietary concentrations of SID Lys above this breakpoint, neither a decrease nor an increase in N utilization was observed. This dietary treatment effect is in agreement with findings of Theil et al. (2002a), who reported greater N retention with a high protein diet compared to a low protein diet on day 104 of gestation. The N utilization is highly affected by stage of gestation due to the changing requirement of the sow (Theil et al., 2002a; Miller et al., 2019; Yang et al., 2022), thus the gestational stage must be similar when comparing studies. On day 104 of gestation, Theil et al. (2002a) reported a maximal N utilization of 55.8% and on day 90 of gestation Yang et al. (2022) reported a N utilization of 64.8%, both being in the range of the present study.

The breakpoint in N utilization indicates that the metabolism of the sow accommodates changes in protein supply. Current findings show that N utilization decreased as dietary SID Lys concentration decreased below the breakpoint of 6.06 SID Lys/kg. The simplest explanation for this decrease in N utilization, is the gradual decrease in N retention as sows are fed decreasing concentrations of SID Lys below the breakpoint, because they are presumably fed in deficit of their high requirement for reproduction, i.e., development of conceptus, mammary glands, and other reproductive tissues, and maintenance. This is evident when considering the reduction in MY below the breakpoint at 5.79 g SID Lys/kg and suggests impaired mammary development. Sows have a remarkable ability to compensate for nutritional deficiencies by mobilizing body reserves. During this process, N from mainly skeletal muscle tissue is mobilized (Clowes et al., 2005) and deposited in the highly prioritized reproductive tissues, including conceptus and mammary glands. However, it is likely that body mobilization cannot completely compensate for the gradual increase in protein deficit, leading to the observed lower MY. Another factor that contributes to the reduction in N utilization is the proportion of the unavoidable cost for maintenance relative to the intake, which increases as N intake decreases, causing N utilization to decrease.

The N balance was determined just before farrowing when the protein requirement of the gestating sow peaks (NRC, 2012). To our knowledge, no studies regarding sows’ protein requirement in the last week of gestation have been conducted and only sparse knowledge exists about the nutritional requirement of the sow in the transition period. According to the factorial approach of Feyera and Theil (2017), the Lys requirement of sows for maintenance and reproduction is approximately 35 g SID Lys/d on day 115 of gestation. This estimated requirement is greater than the daily SID Lys supply of any dietary treatment in the present study (15.2 to 32.6 g SID Lys/d). Considering the identified breakpoint, which was equal to a daily intake of 22 g SID Lys, current findings indicate that the factorial approach used by Feyera and Theil (2017) overestimates multiparous sows’ SID Lys requirement in the last week of gestation.

Despite feeding a wide gradient of dietary protein in the present study, no treatment effects on urine N or urea excretion were identified. We have previously shown that urea production is correlated with protein intake and that plasma urea concentrations exhibit a broken-linear response to dietary protein concentration. When protein is supplied below the sows’ requirement, plasma urea concentrations remain stable due to body protein mobilization whereas when supplying protein in excess of the requirement there is an increase in plasma urea concentration (Hojgaard et al., 2019; Strathe et al., 2020). A similar response was expected under the present experimental settings but was not observed. However, when expressing urinary urea relative to creatinine, a broken-linear relationship with SID Lys concentration became apparent. Assuming that creatinine is excreted at a constant rate dependent mainly on BW (Wyss and Kaddurah-Daouk, 2000) and that the potential contribution from body protein mobilization is neglectable (Strathe et al., 2017a), this broken-linear relationship indicates that urea excretion remains stable until a breakpoint at 6.08 g SID Lys/g, after which it increased along with the dietary concentration of SID Lys, due to oversupply of protein. This urea response is supported by the coinciding breakpoint in the N utilization (6.06 g SID Lys/kg) and results indicate that the sow’s requirement for dietary protein is in the proximity of this breakpoint.

When dietary protein is fed above the requirement for maintenance and reproduction it has one of two fates, depending on the energy status of the sow. If energy is in surplus, excess protein is stored as maternal protein. On the other hand, if the sow is in a neutral or negative energy balance, excess AA is oxidized and urea production and excretion increase (Strathe et al., 2017a; Hojgaard et al., 2019). Considering that N utilization reached a plateau while the N retention kept increasing in the present study, it indicates that excess dietary protein is mainly used for maternal protein deposition because a decrease in utilization would be expected if excess protein was oxidized (Pedersen et al., 2019). This assumption is further supported by the high feed supply, and consequently high energy supply (49 to 50 MJ ME/d), of sows in the present study relative to their estimated requirement for maintenance and reproduction (Feyera and Theil, 2017).

### Farrowing performance and CY

The farrowing process is complex and affected by numerous factors that can be related to the animal, the environment, and nutrition (Oliviero et al., 2010; Feyera et al., 2018). In the present study, a positive linear relationship between stillbirth rate and SID Lys was identified, showing that increasing SID Lys increased stillbirth rate. Litter size and farrowing duration are the main factors affecting the stillbirth rate (Gourley et al., 2020; Sens Junior et al., 2023). In the present study, we did not see dietary treatment effects on farrowing duration; however, it must be kept in mind, that farrowing assistance was applied when birth interval exceeded 1 h, which may have leveled off any potential effect on the length of farrowing. We did, however, find a positive linear relationship between total-born piglets and SID Lys. Nevertheless, this relationship is unexplainable because, the experiment took place in the last week of gestation and piglets dying prepartum in this period would have been registered as stillborn at farrowing, thus still contributing to the number of total-born piglets. To avoid interference of this relationship between total-born piglets and SID Lys on results on the stillbirth rate, the number of total-born piglets was included as an offset in the model for stillbirth rate. Also, in agreement with present study Tydlitát et al. (2008) reported an increase in farrowing duration and stillbirth rate when increasing the dietary protein concentration from 134 g/kg to 210 g/kg. Moreover, Hojgaard et al. (2022) recently showed that increasing the feed supply from 2.8 to 3.6 kg/d, at least three days before farrowing only reduces stillbirth rate if the concentration of SID Lys (protein) is reduced (6.3 vs. 7.6 g/kg of SID Lys). As previously discussed, in the present study excess protein is presumably used for maternal protein deposition which requires energy. Thus, sows fed protein above their requirement would have less energy available, which potentially could have impaired the farrowing process (Feyera et al., 2018, 2021). This positive relationship between stillbirth rate and SID Lys cannot be ignored when determining the optimal concentration of protein in transition diets for sows. Preferentially, the concentration of SID Lys should be kept as low as possible without impairing sow performance excessively.

The sow’s capacity to produce colostrum likely depends on the development of the mammary glands in terms of the number of epithelial cells, however, such relation has never been established (Theil et al., 2022). During late gestation, mammary development is highly accelerated and prone to nutritional manipulation (Farmer and Hurley, 2015). Despite the large influence of colostrum intake on piglet health and growth (Dividich et al., 2005; Decaluwé et al., 2014b), only few studies were conducted and even fewer were able to show any nutritional effects on CY. Among these, Feyera et al. (2021) recently found that CY is maximized at a feed supply of 3.0 kg/d and remains stable at increasing feed supply. Also, Decaluwé et al. (2014a) found a tendency for a decrease in CY at a low feed supply (1.5 vs. 4.5 kg/d) and Wiegert (2019) reported a tendency for increased colostrum intake of piglets with increasing feed levels to sows in transition (1.5 to 4.5 kg). Even though we did not demonstrate the effect of dietary treatment on CY, the average CY observed in the present study (7.0 kg/sow) is in agreement with the findings of Feyera et al. (2021) and Pedersen et al. (2020) in multiparous sows of similar genetics. Current findings therefore show that colostrum production is highly prioritized and that even a severe dietary protein deficit does not affect this process. Considering findings of Feyera et al. (2021) and Decaluwé et al. (2014a) current results further indicate that feed supply has a greater influence on colostrum production than protein supply.

### Carry-over effect on milk production

During the last week of gestation, protein accretion for fetal growth and mammary development is highly accelerated (Feyera and Theil, 2017) and large quantities of AA are taken up by the mammary gland (Krogh et al., 2017). It is well established that mammary development influences MY and any attempt to affect mammogenesis must be conducted when mammary development is already ongoing, such as in late gestation (Farmer, 2018). In the present study, the broken-linear relationship between dietary treatment during transition and MY revealed a breakpoint at 5.79 g SID Lys/kg for a maximal MY of 13.5 kg. This result implies that dietary protein supply during the transition period has a carry-over effect on MY in the subsequent lactation, presumably through mammary development. This effect on MY is also reflected in the ADFI of lactating sows, which tended to increase until a dietary SID Lys concentration of 4.91 g/kg after which it remained steady. Such a relation between MY and sow ADFI was also reported by Strathe et al. (2017b). Present results show that despite the relation between ADFI and MY, there were greater body fat losses with increasing SID Lys concentrations, presumably because of the higher nutrient demand for MY. Litter ADG is the main predictor of MY, which is visualized by the close resemblance of the two linear-broken relationships. However, no significant breakpoint on litter ADG was found in the present study, which indicates that litter size, which is the only other predictor of MY, cannot be neglected for estimating MY of sows (Hansen et al., 2012).

A carry-over effect of feed supply during transition on MY recently reported by Bruun et al. (2023), showed that the lactation performance of sows was maximized when fed 4.1 kg/d during the transition period. This feed supply is equivalent to a daily intake of 32.8 g SID Lys, which is considerably higher than the optimal SID Lys supply for MY (22 g SID Lys/d at 5.79 SID Lys/kg) identified in the present study at a slightly lower feed supply (3.8 kg/d). This demonstrates the importance of optimizing not only the feed intake but also dietary concentration of nutrients.

The mammary development of gilts was shown to be susceptible to dietary manipulations (Farmer, 2018), and recently Farmer et al. (2022) reported enhanced mammary development in gilts that were fed 40% above their SID Lys requirement during late pregnancy as estimated by NRC (2012). Kusina et al. (1999) also observed improved lactation performance when gestating gilts were fed increased dietary protein (4 to 16 g Lys/d). A recent study by Farmer et al. (2023) indicates that such stimulating effect of protein on the mammary development does not apply to multiparous sows, presumably because their mammary gland is already fully developed. On the other hand, Che et al. (2019) observed increased piglet ADG and a tendency of greater weaning weight, when feeding multiparous sows, a high AA diet (20.6 g SID Lys/d) as compared to a low AA diet (14.7 g SID Lys/d) from day 90 of gestation until farrowing. Taking the findings of Farmer et al. (2023) into account, it seems likely that mammary development of sows fed the low AA diet may have been impaired, rather than the high AA diet improved mammary development. Sows have a remarkable ability to compensate for nutritional deficits by mobilizing body reserves, yet it appears that below a certain threshold, the mobilization cannot counterbalance the deficit, and the performance is impaired.

## Conclusion

Results from the present study suggest that the transition diet of multiparous sows fed 3.8 kg/d should contain 5.79 g SID Lys/kg to ensure optimal lactational performance, while a dietary concentration of 6.06 g SID Lys/kg is required for also optimizing N utilization in the transition period. Due to the detrimental effect of increasing SID Lys on stillbirth rate, the concentration of SID Lys should be kept as low as possible without impairing sow performance excessively. Feeding below 5.79 g SID Lys/kg during transition impaired MY in the subsequent lactation, presumable due to reduced mammary development, and caused a higher mobilization of body protein. Dietary concentration of SID Lys did not affect CY underlining the importance of energy rather than protein for this process. Present study demonstrates the importance of supplying dietary protein in accordance with the requirement of the sow, to avoid detrimental effects of inadequate and excess protein supply. In conclusion, the transition diet of multiparous sows should contain 5.79 g SID Lys/kg when fed 3.8 kg/d (13.0 MJ ME/kg), for a total SID Lys intake of 22 g/d.

## Abbreviations

AA: amino acid
ADG: average daily gain
ATTD: apparent total tract digestibility
BF: back fat
BW: body weight
CP: crude protein
CY: colostrum yield
D_2_O: deuterium oxide
Lys: lysine
MY: milk yield
N: nitrogen
NEFA: nonesterified fatty acid
SID: standardized ileal digestible
TiO_2_: titanium dioxide
TG: triglycerides
